# Thalamic haemorrhage vs internal capsule-basal ganglia haemorrhage: clinical profile and predictors of in-hospital mortality

**DOI:** 10.1186/1471-2377-7-32

**Published:** 2007-10-05

**Authors:** Adrià Arboix, Raquel Rodríguez-Aguilar, Montserrat Oliveres, Emili Comes, Luis García-Eroles, Joan Massons

**Affiliations:** 1 Cerebrovascular Division, Department of Neurology, Hospital Universitari del Sagrat Cor, Universitat de Barcelona, Barcelona, Spain; 2 Department of Internal Medicine, Hospital Universitari del Sagrat Cor, Universitat de Barcelona, Barcelona, Spain; 3 Clinical Information Systems, Hospital Universitari de Bellvitge, L'Hospitalet de Llobregat, Barcelona, Spain

## Abstract

**Background:**

There is a paucity of clinical studies focused specifically on intracerebral haemorrhages of subcortical topography, a subject matter of interest to clinicians involved in stroke management. This single centre, retrospective study was conducted with the following objectives: a) to describe the aetiological, clinical and prognostic characteristics of patients with thalamic haemorrhage as compared with that of patients with internal capsule-basal ganglia haemorrhage, and b) to identify predictors of in-hospital mortality in patients with thalamic haemorrhage.

**Methods:**

Forty-seven patients with thalamic haemorrhage were included in the "Sagrat Cor Hospital of Barcelona Stroke Registry" during a period of 17 years. Data from stroke patients are entered in the stroke registry following a standardized protocol with 161 items regarding demographics, risk factors, clinical features, laboratory and neuroimaging data, complications and outcome. The region of the intracranial haemorrhage was identified on computerized tomographic (CT) scans and/or magnetic resonance imaging (MRI) of the brain.

**Results:**

Thalamic haemorrhage accounted for 1.4% of all cases of stroke (*n *= 3420) and 13% of intracerebral haemorrhage (*n *= 364). Hypertension (53.2%), vascular malformations (6.4%), haematological conditions (4.3%) and anticoagulation (2.1%) were the main causes of thalamic haemorrhage. In-hospital mortality was 19% (*n *= 9). Sensory deficit, speech disturbances and lacunar syndrome were significantly associated with thalamic haemorrhage, whereas altered consciousness (odds ratio [OR] = 39.56), intraventricular involvement (OR = 24.74) and age (OR = 1.23), were independent predictors of in-hospital mortality.

**Conclusion:**

One in 8 patients with acute intracerebral haemorrhage had a thalamic hematoma. Altered consciousness, intraventricular extension of the hematoma and advanced age were determinants of a poor early outcome.

## Background

Most studies of primary intracerebral haemorrhages are focused on the global assessment of patients with haemorrhagic stroke independent of the different topography of lesions [1,2]. It has recently been shown that the clinical spectrum, prognosis and early mortality of patients with primary intracerebral haemorrhage are reasonably dependent on the site of bleeding [3]. On the other hand, thalamic haemorrhages and basal ganglia-internal capsule haemorrhages are a group of supratentorial cerebral haemorrhages of subcortical topography, with clinical characteristics that are clearly different from those of the remaining cases of lobar or brainstem haemorrhages [1,2]. However, the differential clinical profile between subcortical cerebral haemorrhages of isolated thalamic topography and haemorrhages involving the basal ganglia and the internal capsule is poorly defined. Moreover, predictors of in-hospital mortality in patients with isolated thalamic haemorrhage are not clearly established probably because in clinical series collected from hospital-based stroke registries, separate analysis of thalamic haemorrhage as an individual clinical entity is rarely performed.

This aim of this study was to describe the aetiological, clinical and prognostic characteristics of patients with thalamic haemorrhage as compared with that of patients with basal ganglia-internal capsule haemorrhage collected from a prospective hospital-based stroke registry. A second objective was to identify predictors of in-hospital mortality in patients with thalamic haemorrhage.

## Methods

The database of the "Sagrat Cor Hospital of Barcelona Stroke Registry" with data of 3420 acute stroke patients was searched for those with a diagnosis of intracerebral haemorrhage who were admitted consecutively to the Department of Neurology of the Sagrat Cor Hospital (an acute-care 350-bed teaching hospital in the city of Barcelona) between January 1986 and December 2002. Details of this on-going hospital-based stroke registry have been reported in previous studies [4,5]. Data from stroke patients are entered following a standardized protocol with 161 items regarding demographics, risk factors, clinical features, laboratory and neuroimaging data, complications and outcome. Subtypes of stroke were classified according to the Cerebrovascular Study Group of the Spanish Neurological Society [6], which is similar to the National Institute of Neurological Disorders and Stroke Classification [7]. Definitions of cerebrovascular risk factors have been used by our group in previous studies [3-5].

For the purpose of this study, primary intracerebral haemorrhages diagnosed in 364 patients were collected. The region of the intracranial haemorrhage was identified on computerized tomographic (CT) scans and/or magnetic resonance imaging (MRI) of the brain. The volume of haematomas was measured. Haematomas were divided into small and large using a diameter of 30 mm. Topographies included the thalamus in 47 patients, internal capsule and basal ganglia in 77, cerebral lobes in 140, cerebellum in 27, brainstem in 18, primary intraventricular haemorrhage in 11 and multiple topographic involvement (when more than one of the aforementioned topographies was affected by the haemorrhage and the size of the haematoma was > 30 mm) in 44. The objective of this clinical study was to assess differential features in aetiology, risk factors, clinical findings and early outcome between the cohorts of patients with thalamic haemorrhage (*n *= 47) and internal capsule-basal ganglia haemorrhage (*n *= 77). Prior to conducting the study, approval was obtained from the Ethical Committee of Clinical Research of the hospital.

All patients were admitted to the hospital within 48 hours of onset of symptoms. On admission, demographic characteristics; salient features of clinical and neurological examination and results of laboratory tests (blood cell count, biochemical profile, serum electrolytes, urinalysis); chest radiography; twelve-lead electrocardiography; and brain CT and/or MRI were recorded. Angio-MRI was obtained during hospitalisation in 17% of cases and arterial digital subtraction angiography was performed in 16% of patients with thalamic haemorrhage in whom we could not find any risk factor for haemorrhagic stroke. Other investigations included echocardiography in 10.6% of patients and lumbar puncture in 6.4%. Degree of clinical disability at discharge from the hospital was evaluated according to modified Rankin scale (mRS) [8], and causes of death according to the criteria of Silver et al. [9].

### Statistical analysis

Demographic characteristics, risk factors, clinical events and outcome of patients with thalamic haemorrhage and those with internal capsule-basal ganglia haemorrhage were compared using the Student's *t*-test or the Mann-Whitney U test for continuous variables and the chi-square (χ^2^) test (with Yate's correction when necessary) for categorical variables. Variables were subjected to multivariate analysis with a logistic regression procedure and forward stepwise selection if *P *< 0.10 after univariate testing. The effect of variables on the presence of thalamic haemorrhage and fatal outcome was studied in two multiple regression models based on demographic, vascular risk factors, and clinical and neuroimaging variables, in which thalamic haemorrhage and in-hospital mortality were the dependent variables, respectively. Statistical significance was set at *P *< 0.05.

## Results

Thalamic haemorrhage accounted for 1.4% of all cases of stroke (*n *= 3420) and 13% of intracerebral haemorrhage (*n *= 364). There were 27 men and 20 women with a mean (SD) age of 71.6 (10.9) years. The following vascular risk factors in a decreasing order of frequency were observed: history of hypertension (61.7%), diabetes mellitus (17%) and alcohol abuse (> 80 g/day) (12.8%). Sudden onset of neurological deficit was recorded in 74.5% of cases. Motor deficit occurred in 78.7% of cases, sensory deficit in 70.2%, altered consciousness in 31.9%, headache in 29.8%, nausea or vomiting in 27.7%, speech disturbances (dysarthria, aphasia) in 21.3% and oculomotor and visual disturbances in 21.3% (upward gaze palsy with miotic unreactive pupils, skew ocular deviation). Abnormal involuntary movements were present in only 2 patients. On the other hand, 4 patients had a lacunar syndrome (pure sensory stroke 3, sensorimotor stroke 1). The topography of thalamic lesion [10] was anterior in 6% of cases, posteromedial in 24%, posterolateral in 48%, dorsal in 2% and affected all thalamic vascular territories in 20%. Intraventricular involvement was present in 42.6% of patients.

Hypertension (53.2%), vascular anomaly (6.4%), haematological conditions (4.3%) and anticoagulation (2.1%) were the main causes of thalamic haemorrhage. The aetiology of thalamic bleeding was not identified in 34% of patients. One patient had surgical intervention (ventricular drainage). Median duration of hospital stay was 20 days (25th–75th percentile, 11–30 days). At the time of hospital discharge, 1 patient (2.1%) was symptom free (mRS grade 0). Of the remaining 37 patients, 11 had moderate disability (mRS grade 3), 15 moderately severe disability (mRS grade 4) and 11 severe disability (mRS grade 5). Significant altered memory function was present in 1 patient at hospital discharge. A total of 9 patients died (in-hospital mortality rate 19.1%). The median time of death was 11 days (25th–75th percentile, 6–13.5 days). Cumulative in-hospital mortality in relation to length of hospital stay showed that 33% of all deaths had occurred on day 7, 88.5% on day 14, and 100% on day 90. Causes of death included cerebral herniation in 4 patients, pneumonia in 2, sepsis in 2 and unknown cause in 1.

When the groups of patients with thalamic haemorrhage with favourable and unfavourable outcome were compared (Table 1), advanced age, altered consciousness, neurological complications and intraventricular extension of the haemorrhage were significantly more frequent in patients who died. In the multivariate analysis, advanced age, altered consciousness and intraventricular involvement were independent variables significantly associated with in-hospital death (Table 2).

**Table 1:** Results of univariate analysis in 47 patients with thalamic haemorrhage according to early outcome

Data	Alive (*n *= 38)	Dead (*n *= 9)	*P *value
Age, years, mean (SD)	69.5 (10.9)	80.2 (5.2)	0.002
Sex			
Male	24 (88.9)	3 (11.1)	0.106
Female	14 (70)	6 (30)	0.371
Valvular heart disease			
Absent	38 (100)	8 (17.4)	0.191
Present	0	1 (100)	
Transient ischaemic attack			
Absent	38 (100)	8 (17.4)	0.191
Present	0	1 (100)	
Obesity			
Absent	36 (83.7)	7 (16.7)	0.160
Present	2 (50)	1 (50)	
Hyperlipidemia			
Absent	34 (85)	6 (15)	0.117
Present	4 (57.1)	3 (42.9)	
Nausea, vomiting			
Absent	29 (85.3)	5 (14.7)	0.198
Present	9 (69.2)	4 (30.8)	
Altered consciousness			
Absent	30 (93.8)	2 (6.3)	0.002
Present	8 (53.3)	7 (46.7)	
Sensory deficit			
Absent	9 (64.3)	5 (35.7)	0.074
Present	29 (87.9)	4 (12.1)	
Lacunar syndrome			
Absent	34 (79.1)	9 (20.9)	0.724
Present	4 (100)	0	
Intraventricular involvement			
Absent	26 (96.3)	1 (3.7)	0.003
Present	12 (60)	8 (40.5)	
Neurological complications			
Absent	36 (90)	4 (10)	0.001
Present	2 (28.6)	5 (71.4)	
Respiratory events			
Absent	33 (84.6)	6 (15.4)	0.167
Present	5 (62.5)	3 (37.5)	

Data are *n *(%) unless otherwise stated.

**Table 2:** Variables associated with in-hospital death in patients with thalamic haemorrhage

Variable	β	SE (β)	Odds ratio (95% CI)	*p*
Model based on demographics, vascular risk factors and clinical variables*				
Altered consciousness	3.223	1.120	25.10 (2.8–225.3)	0.004
Age	0.160	0.069	1.17 (1.03–1.34)	0.020
Model based on demographics, vascular risk factors, clinical features and neuroimaging variables^†^				
Altered consciousness	3.678	1.471	39.59 (2.51–707.37)	0.012
Intraventricular involvement	3.209	1.600	24.74 (1.07–569.60)	0.045
Age	0.210	0.103	1.23 (1.01–1.51)	0.042

*^β ^= -15.114; SE (β) = 5.651; goodness-of-fit χ^2 ^= 1.265; *df *= 7; *P *= 0.989; area under the ROC curve = 0.924; sensitivity 60%; specificity 71%; positive predictive value 56%; negative predictive value 74%; correct classification 66.9%.

^†^β = -21.386; SE (β) = 9.304; goodness-of-fit χ^2 ^= 1.604 ; df = 7 ; *P *= 0.978; area under the ROC curve = 0.949; sensitivity 78%; specificity 87%; positive predictive value 57%; negative predictive value 94%; correct classification 85.1%.

The characteristics of patients with thalamic haemorrhage in comparison with the cohort of 77 patients with internal capsule-basal ganglia haemorrhage are shown in Table 3. History of chronic liver disease, sensory symptoms, nausea and/or vomiting and ataxia was significantly more frequent in patients with thalamic haemorrhage, whereas speech disturbances (dysarthria, aphasia) and clinical presentation as a lacunar syndrome were significantly more common among patients with internal capsule-basal ganglia haemorrhage. In the multivariate analysis sensory deficit (odds ratio [OR] = 2.33, 95% confidence interval [CI] 1.10–4.84; *P *= 0.040), speech disturbances (OR = 0.34, 95% CI 0.16–0.86; *P *= 0.015) and lacunar syndrome (OR = 0.26, 95% CI 0.15–0.89; *P *= 0.026) were independent predictors of isolated thalamic haemorrhage.

**Table 3:** Differences between patients with thalamic haemorrhage and patients with internal capsule-basal ganglia haemorrhage

Data	Thalamic haemorrhage	Internal capsule-basal ganglia haemorrhage	*P *value
Total patients	47	77	
Sex, male	27 (57.4)	50 (64.9)	0.259
Age, years, mean (SD)	71.6 (10.9)	72.2 (12.2)	0.661
Vascular risk factors			
Hypertension	29 (61.7)	57 (74)	0.107
Diabetes mellitus	8 (17)	9 (11.7)	0.282
Dyslipemia	7 (14.9)	10 (13)	0.481
Atrial fibrillation	5 (10.6)	11 (14.3)	0.384
Ischaemic heart disease	3 (6.4)	5 (6.5)	0.646
Cigarette smoking (> 20/day)	3 (6.4)	8 (10.4)	0.339
Chronic liver disease	5 (10.6)	0	0.007
Clinical findings			
Sudden onset (min)	35 (74.5)	48 (52.3)	0.115
Limb weakness	37 (78.7)	67 (87)	0.167
Sensory symptoms	33 (70.2)	38 (49.4)	0.018
Altered consciousness	15 (31.9)	23 (29.9)	0.482
Headache	14 (29.8)	18 (23.4)	0.279
Nausea, vomiting	13 (27.7)	10 (13)	0.037
Speech disturbances (dysarthria, aphasia)	10 (21.3)	34 (44.2)	0.008
Ataxia	4 (8.5)	0	0.019
Lacunar syndrome	4 (8.5)	18 (23.4)	0.036
Ventricular involvement	20 (42.6)	12 (15.6)	0.001
Outcome			
In-hospital mortality	9 (19.1)	13 (16.9)	0.464
Respiratory complications	8 (17)	8 (10.4)	0.212
Urinary complications	8 (17)	7 (9.1)	0.152
Infectious complications	16 (34)	17 (22.1)	0.106
Symptom-free at discharge	1 (2.1)	6 (7.8)	0.180

Data are *n *(%) unless otherwise stated.

## Discussion

Data regarding the frequency of isolated thalamic haemorrhage in the different hospital-based stroke registries are scarce, the present results show that thalamic haematomas is a subgroup of haemorrhagic stroke that accounted for 1.4% of all cases of stroke and 13% of intracerebral haemorrhages. The prevalence of thalamic haemorrhage in different series of primary intracerebral haemorrhage vary widely from 6% in the series of Juvela et al. [11] to 15.7% in the series of Tatu et al. [12]. In a subsample of 390 with haemorrhagic stroke reported by Kumral et al. [13], thalamic haemorrhage was diagnosed in 100 patients (25.6%).

Results of the present study show that patients with thalamic haemorrhage and patients with internal capsule-basal ganglia haemorrhage presented different clinical profiles, with sensory disturbances being significantly more frequent and speech disorders and lacunar syndrome being significantly less frequent in patients with thalamic haematoma. Prominent sensory loss, either anaesthesia or hypaesthesia affecting face, limbs and trunk, generally for all sensory modalities was found in 70.2% of patients, which is consistent with classical reports showing the predominance of sensory deficit as a cardinal feature of thalamic haemorrhage especially when the ventroposterolateral nucleus is affected [1,14,15].

Speech disturbances are less frequent in patients with thalamic haemorrhage (21%) as compared with those with internal capsule-basal ganglia haemorrhage (44.2%). Dysarthria and different types of aphasia was explained in thalamic haemorrhage as a disruption of any circuit (arranged as frontal rostrocaudal/thalamic mediolaterally) leading to dysfunction [1,2]. However, interruption of the corticolingual pathways in the internal capsule may explain the higher frequency of speech disturbances (mainly dysarthria) in internal capsule-basal ganglia haemorrhages [16,17].

Clinical presentation of thalamic haemorrhage in the form of a lacunar syndrome is very rare, and was documented in only 4 patients (8.5%), 3 of whom presenting with a pure sensory stroke and 1 a sensorimotor syndrome. This finding is in accordance with a recent study of our group [18] and with data reported in the literature showing that pure sensory stroke is classically associated with a lacunar thalamic infarction [19].

Whereas none of the patients in the series of Kumral et al. [13] presented with pure sensorimotor stroke, a lacunar syndrome was observed in 23.4% of our patients with internal capsule-basal ganglia haemorrhage, mainly in the form of a pure motor stroke or sensorimotor stroke. These findings are consistent with the study of Weisberg and Wall (20) who reported pure motor hemiparesis in 7 out of 10 patients as well as with other reports indicating that a small haematoma of subcortical topography in the internal capsule but also in the basal ganglia may cause a lacunar syndrome [21].

Thalamic haemorrhage is a severe clinical condition with an in-hospital mortality rate, in the present study, of 19%, and only one patient (2.1%) was symptom-free at discharge. The mortality rate of thalamic haemorrhage was 12% after 6 ± 6 days after stroke and 17.3% within 6 months in the series of Mori et al. [22]. In the series of Chung et al. [23], the case fatality was 37% at the time of discharge. On the other hand, the mortality rate of thalamic haemorrhage is generally lower than that of brainstem haemorrhages or cerebral haemorrhages of multiple topographies, which show a very high in-hospital mortality rate usually greater than 40% [3]. The mortality rate in patients with thalamic haemorrhage, however, is higher than that of patient with capsular stroke [3,16,17]. Altered consciousness, intraventricular hemorrhage and advanced age were independent predictors of in-hospital mortality in patients with thalamic haematoma.

The initial level of consciousness was always found to be a predictor of mortality in the differents series [3,11-15]. Previous studies reported that initially comatose or stupurous patients had the poorest chance of survival [1,2]. Intraventricular haemorrhage has been found to be a predictor of in-hospital mortality in some studies [13,24,25] but not in others [11,22,26] and one study has even suggested that intraventricular bleeding is associated with improved outcome in thalamic haemorrhage [27]. However, our results shows that stroke-related deaths occurred in 40.5% of intraventricular haemorrhage cases and agree with that of the Steinke et al. [25] who found that in-hospital mortality occurred in 52% of thalamic haematomas with ventricular extension and that intraventricular extension is an powerful independent predictor of mortality (Table 4). Death increased with age and agree with the study of Mori et al. [22]. In the study of Daverat el al. [28], age was the most important predictor of death and functional outcome after spontaneous intracerebral haemorrhage.

**Table 4:** Thalamic haemorrhage. Series reported in the literature

Author, year [reference]	Patients	Type of study	Frequency intracerebral haemorrhages (%)	Ventricular Extension (%)	Ventricular extension as predictor of in-hospital death	In-hospital mortality (%)
Walshe, 1977 [14]	18	Clinical series		66		
Barraquer-Bordas, 1981 [15]	23	Clinical series		50		39
Kwak, 1983 [26]	29	Clinical series			No	
Weisberg, 1986 [24]	50	Clinical series		38	Yes	38
Steinke, 1992 [25]	44	Stroke data bank		47.7	Yes	31.8
Kumral, 1995 [13]	100	Stroke data bank	25.6	57	Yes	25
Mori, 1995 [22]	104	Clinical series		44	No	17.3 (6 months)
Lampl, 1995 [27]*	52	Clinical series		63.5	No	36.6 (6 moths)
Chung, 1996 [23]	175	Clinical series			^†^	37
Shah, 2005 [30]	53	Clinical series		66.6	No	15.7
Present series, 2007	47	Hospital-based stroke registry	13	42.6	Yes	19.1

*11 patients with thalamo-capsular haemorrhages and 41 patients with isolated thalamic haemorrhages

^†^Presence of dense blood clot in the third ventricle in a CT scan indicate a poor outcome

Early expansion of cerebral haematoma of any topography is an important determinant of in-hospital mortality [29,30]. Recent studies have shown that ultra-early haemostatic therapy with recombinant activated factor VII (rFVIIa) within four hours after the onset of intracerebral hemorrhage limits the growth of the haematoma, reduces mortality, and improves functional outcomes [31]. These preliminary findings will probably determine a change in the management of patients with acute intracerebral haemorrhage, in which ultra-early haemostatic therapy, – similar to the efficacy of early thrombolytic therapy in cerebral infarction –, followed by careful monitorization in specialised stroke units could be of paramount importance in the care of patients with intracerebral haemorrhage [32].

## Conclusion

Approximately one in every 8 patients with acute intracerebral haemorrhage had a thalamic haematoma. Patients with isolated thalamic haemorrhage show a differential clinical profile than patients with internal capsule-basal ganglia haemorrhage. Altered consciousness, intraventricular involvement and advanced age were independent predictors of in-hospital mortality.

## Abbreviations

CI: Confidence interval.

CT: Computed tomography

MRI: Magnetic resonance imaging

OR: Odds ratio.

ROC: Receiver operating characteristics.

SE: Standard error.

## Competing interests

The author(s) declare that they have no competing interests.

## Authors' contributions

AA was the principal investigator, chief of the Cerebrovascular Division, designed the study, diagnosed and took care of the patients, contributed to analyze the data, interpreted the results, wrote the paper, and prepared the final draft. He was also responsible for editorial decisions including the selection of the journal.

RR participated in the collection of data, search and review of the literature, analysis of results, review of the manuscript, and approved the final draft.

LG-E was the statistician, participated in the study design, analysis and interpretation of data, wrote the part of the paper related to the statistical analysis, and approved the final draft.

EC, JM, and MO diagnosed and took care f the patients, contributed in the review of the literature, interpretation of the results, review of the paper for intellectual content, and approved the final draft.

## Pre-publication history

The pre-publication history for this paper can be accessed here:


